# Tetra­kis(triphenyl­phosphane-κ*P*)silver(I) tetra­fluorido­borate

**DOI:** 10.1107/S1600536812018247

**Published:** 2012-04-28

**Authors:** Xu Huang, Qi-Ming Qiu, Xin Wang, Qiong-Hua Jin, Cun-Lin Zhang

**Affiliations:** a Department of Chemistry, Capital Normal University, Beijing 100048, People’s Republic of China; b Key Laboratory of Terahertz Optoelectronics, Ministry of Education, Department of Physics, Capital Normal University, Beijing 100048, People’s Republic of China

## Abstract

The title complex, [Ag(C_18_H_15_P)_4_]BF_4_, was prepared by the reaction of silver(I) tetra­fluorido­borate and triphenyl­phosphane in the presence of 1,2-bis­(pyridin-2-yl)ethyl­ene. The Ag^I^ atom is tetra­hedrally coordinated by four P atoms from triphenyl­phosphane (PPh_3_) ligands. Due to symmetry, the tetra­fluorido­borate anion is disordered over three positions (each with one third occupancy). The tetra­fluorido­borate anion does not coordinate to the Ag^I^ atom.

## Related literature

For background to silver(I) complexes, see: Bowmaker *et al.* (1993 ▶); Cui, Hu *et al.* (2010 ▶); Cui, Jin *et al.* (2010 ▶); Jin, Hu *et al.* (2010 ▶); Jin, Song *et al.* (2010 ▶); Meijboom *et al.* (2009 ▶). For related structures, see: Engelhardt *et al.* (1985 ▶); Wen *et al.* (2011 ▶).
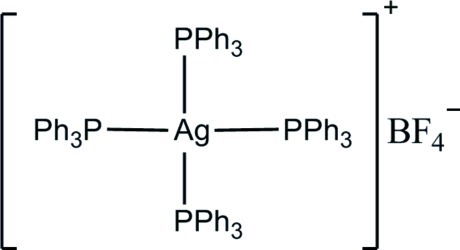


## Experimental

### 

#### Crystal data

[Ag(C_18_H_15_P)_4_]BF_4_
*M* *_r_* = 1243.76
Trigonal, 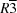

*a* = 14.2445 (12) Å
*c* = 51.591 (4) Å
*V* = 9065.7 (12) Å^3^
*Z* = 6
Mo *K*α radiation
μ = 0.50 mm^−1^
*T* = 298 K
0.30 × 0.18 × 0.15 mm

#### Data collection

Bruker SMART 1000 CCD diffractometer
Absorption correction: multi-scan (*SADABS*; Bruker, 2007 ▶) *T*_min_ = 0.866, *T*_max_ = 0.930
15472 measured reflections
3548 independent reflections
2167 reflections with *I* > 2σ(*I*)
*R*_int_ = 0.092

#### Refinement

*R*[*F* ^2^ > 2σ(*F* ^2^)] = 0.063
*wR*(*F* ^2^) = 0.160
*S* = 1.07
3548 reflections
272 parameters
1 restraint
H-atom parameters constrained
Δρ_max_ = 1.34 e Å^−3^
Δρ_min_ = −0.52 e Å^−3^

### 

Data collection: *SMART* (Bruker, 2007 ▶); cell refinement: *SAINT-Plus* (Bruker, 2007 ▶); data reduction: *SAINT-Plus*; program(s) used to solve structure: *SHELXS97* (Sheldrick, 2008 ▶); program(s) used to refine structure: *SHELXL97* (Sheldrick, 2008 ▶); molecular graphics: *SHELXTL* (Sheldrick, 2008 ▶); software used to prepare material for publication: *SHELXTL*.

## Supplementary Material

Crystal structure: contains datablock(s) global, I. DOI: 10.1107/S1600536812018247/ez2286sup1.cif

Structure factors: contains datablock(s) I. DOI: 10.1107/S1600536812018247/ez2286Isup2.hkl

Additional supplementary materials:  crystallographic information; 3D view; checkCIF report

## Figures and Tables

**Fig. 1.**
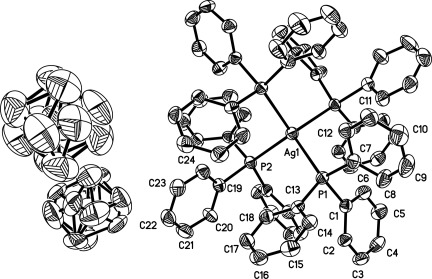
The molecular entities of the title compound, showing the atom-numbering scheme and with displacement ellipsoids drawn at the 50% probability level.

## supplementary crystallographic information

### Comment

The coordination chemistry of silver(I) is of considerable interest because of the luminescence properties and potential applications of silver(I) complexes in catalysis, cyanide, photography antimicrobial activities and electrochemical processes (Bowmaker *et al.*, 1993; Cui, Hu *et al.*, 2010; Cui, Jin *et al.*, 2010; Jin, Hu *et al.*,2010; Jin, Song *et al.*, 2010; Meijboom *et al.*, 2009;). In our previous work, we obtained an Ag^I^complex containing triphenylphosphane and trifluoromethanesulfonate ligands [Ag(PPh_3_)_4_(SO_3_CF_3_)](Wen *et al.*, 2011). In this paper, we report a similar new silver(I) compound [Ag(P(C_6_H_5_)_3_)_4_][(BF_4_)].

The title compound is isomorphous with both [Ag(PPh_3_)_4_]ClO_4_ (Engelhardt *et al.*, 1985) and [Ag(PPh_3_)_4_](SO_3_CF_3_) (Wen *et al.*, 2011). The silver atom is coordinated by four P atoms from four triphenylphosphane ligands to form a tetrahedral geometry around the silver atom.

In [Ag(P(C_6_H_5_)_3_)_4_][(BF_4_)], the Ag—P bond lengths are in the range 2.633 (2)–2.6774 (13) Å, which agrees with those in the compound [Ag(PPh_3_)_4_(SO_3_CF_3_)] (Wen *et al.*, 2011). The distorted P—Ag—P bite angles [P2—Ag1—P1 = 109.45 (3)°, P1—Ag1—P1 = 109.49 (3)°] are similar to the corresponding values for [Ag(PPh_3_)_4_(SO_3_CF_3_)] (Wen *et al.*, 2011), too.

### Experimental

A mixture of silver(I) tetrafluoridoborate and triphenylphosphane (PPh_3_) (molar ratio 1:1) and 1,2-di(2-pyridyl)ethylene (0.2 mmol) in the mixed solution of CH_3_OH (5 ml)/CH_2_Cl_2_ (5 ml) was stirred for 6 h at ambient temperature. The insoluble residues were removed by filtration, and the filtrate was evaporated slowly at room temperature for about three days to yield colorless crystals. Crystals suitable for single-crystal X-ray diffraction were selected directly from the sample as prepared.

### Refinement

H-atoms were positioned and refined using a riding model, with aromatic C—H = 0.93 Å, and the displacement parameters set for phenyl, H atoms at *U*_iso_(H)=1.2*U*_eq_(C).

### Crystal data

**Table:**

[Ag(C_18_H_15_P)_4_]BF_4_	*D*_x_ = 1.367 Mg m^−^^3^
*M**_r_* = 1243.76	Mo *K*α radiation, λ = 0.71073 Å
Trigonal, *R*3	Cell parameters from 2109 reflections
*a* = 14.2445 (12) Å	θ = 2.4–18.9°
*c* = 51.591 (4) Å	µ = 0.50 mm^−^^1^
*V* = 9065.7 (12) Å^3^	*T* = 298 K
*Z* = 6	White, block
*F*(000) = 3840	0.30 × 0.18 × 0.15 mm

### Data collection

**Table:**

Bruker SMART 1000 CCD diffractometer	3548 independent reflections
Radiation source: fine-focus sealed tube	2167 reflections with *I* > 2σ(*I*)
Graphite monochromator	*R*_int_ = 0.092
phi and ω scans	θ_max_ = 25.0°, θ_min_ = 2.3°
Absorption correction: multi-scan (*SADABS*; Bruker, 2007)	*h* = −16→16
*T*_min_ = 0.866, *T*_max_ = 0.930	*k* = −15→16
15472 measured reflections	*l* = −61→40

### Refinement

**Table:**

Refinement on *F*^2^	Primary atom site location: structure-invariant direct methods
Least-squares matrix: full	Secondary atom site location: difference Fourier map
*R*[*F*^2^ > 2σ(*F*^2^)] = 0.063	Hydrogen site location: inferred from neighbouring sites
*wR*(*F*^2^) = 0.160	H-atom parameters constrained
*S* = 1.07	*w* = 1/[σ^2^(*F*_o_^2^) + (0.0699*P*)^2^] where *P* = (*F*_o_^2^ + 2*F*_c_^2^)/3
3548 reflections	(Δ/σ)_max_ = 0.001
272 parameters	Δρ_max_ = 1.34 e Å^−^^3^
1 restraint	Δρ_min_ = −0.52 e Å^−^^3^

### Special details

**Table:**

Geometry. All e.s.d.'s (except the e.s.d. in the dihedral angle between two l.s. planes) are estimated using the full covariance matrix. The cell e.s.d.'s are taken into account individually in the estimation of e.s.d.'s in distances, angles and torsion angles; correlations between e.s.d.'s in cell parameters are only used when they are defined by crystal symmetry. An approximate (isotropic) treatment of cell e.s.d.'s is used for estimating e.s.d.'s involving l.s. planes.
Refinement. Refinement of *F*^2^ against ALL reflections. The weighted *R*-factor *wR* and goodness of fit *S* are based on *F*^2^, conventional *R*-factors *R* are based on *F*, with *F* set to zero for negative *F*^2^. The threshold expression of *F*^2^ > σ(*F*^2^) is used only for calculating *R*-factors(gt) *etc*. and is not relevant to the choice of reflections for refinement. *R*-factors based on *F*^2^ are statistically about twice as large as those based on *F*, and *R*- factors based on ALL data will be even larger.

### Fractional atomic coordinates and isotropic or equivalent isotropic displacement parameters (Å^2^)

**Table:**

	*x*	*y*	*z*	*U*_iso_*/*U*_eq_	Occ. (<1)
Ag1	0.6667	0.3333	0.081276 (13)	0.0476 (3)	
P1	0.80962 (10)	0.53162 (10)	0.06400 (3)	0.0464 (4)	
P2	0.6667	0.3333	0.13231 (4)	0.0475 (6)	
F1	0.364 (3)	0.600 (2)	0.1780 (6)	0.112 (6)	0.33
F2	0.273 (4)	0.604 (2)	0.1863 (5)	0.166 (11)	0.33
F3	0.021 (3)	0.921 (2)	0.0089 (5)	0.140 (6)	0.33
F4	−0.023 (4)	1.036 (3)	0.0208 (3)	0.146 (9)	0.33
B1	0.3333	0.6667	0.1667	0.113 (9)	
B2	0.0000	1.0000	0.0000	0.130 (10)	
C1	0.9477 (4)	0.5677 (4)	0.07293 (10)	0.0490 (13)	
C2	1.0219 (4)	0.6654 (4)	0.08427 (11)	0.0575 (14)	
H2	1.0038	0.7192	0.0865	0.069*	
C3	1.1221 (5)	0.6841 (5)	0.09236 (13)	0.0707 (17)	
H3	1.1708	0.7502	0.0999	0.085*	
C4	1.1502 (5)	0.6060 (5)	0.08940 (13)	0.0742 (18)	
H4	1.2170	0.6179	0.0953	0.089*	
C5	1.0794 (5)	0.5100 (5)	0.07767 (14)	0.0774 (19)	
H5	1.0994	0.4578	0.0749	0.093*	
C6	0.9783 (5)	0.4904 (5)	0.06992 (12)	0.0702 (17)	
H6	0.9299	0.4239	0.0625	0.084*	
C7	0.8161 (4)	0.5488 (4)	0.02889 (11)	0.0566 (15)	
C8	0.9121 (5)	0.6150 (6)	0.01588 (12)	0.083 (2)	
H8	0.9780	0.6462	0.0246	0.099*	
C9	0.9082 (6)	0.6344 (7)	−0.01077 (15)	0.111 (3)	
H9	0.9714	0.6824	−0.0195	0.133*	
C10	0.8122 (7)	0.5829 (7)	−0.02380 (14)	0.097 (2)	
H10	0.8104	0.5951	−0.0415	0.117*	
C11	0.7195 (6)	0.5142 (6)	−0.01119 (13)	0.0782 (18)	
H11	0.6550	0.4776	−0.0204	0.094*	
C12	0.7192 (5)	0.4979 (5)	0.01485 (11)	0.0648 (16)	
H12	0.6543	0.4528	0.0233	0.078*	
C13	0.8024 (4)	0.6496 (4)	0.07541 (10)	0.0486 (13)	
C14	0.8377 (4)	0.7441 (4)	0.06072 (12)	0.0626 (15)	
H14	0.8596	0.7463	0.0436	0.075*	
C15	0.8401 (5)	0.8340 (5)	0.07156 (14)	0.0731 (18)	
H15	0.8654	0.8973	0.0619	0.088*	
C16	0.8049 (5)	0.8295 (5)	0.09661 (15)	0.0754 (19)	
H16	0.8075	0.8905	0.1039	0.090*	
C17	0.7665 (5)	0.7372 (5)	0.11083 (12)	0.0705 (17)	
H17	0.7405	0.7345	0.1275	0.085*	
C18	0.7660 (4)	0.6478 (5)	0.10054 (11)	0.0612 (15)	
H18	0.7410	0.5854	0.1105	0.073*	
C19	0.6357 (4)	0.4300 (4)	0.14761 (10)	0.0493 (13)	
C20	0.6937 (5)	0.4944 (5)	0.16853 (11)	0.0652 (16)	
H20	0.7538	0.4923	0.1749	0.078*	
C21	0.6615 (6)	0.5616 (5)	0.17986 (13)	0.085 (2)	
H21	0.6982	0.6021	0.1944	0.101*	
C22	0.5770 (6)	0.5693 (5)	0.17011 (14)	0.0799 (19)	
H22	0.5571	0.6161	0.1777	0.096*	
C23	0.5210 (5)	0.5077 (5)	0.14904 (13)	0.0760 (18)	
H23	0.4633	0.5131	0.1423	0.091*	
C24	0.5495 (5)	0.4394 (5)	0.13809 (12)	0.0641 (16)	
H24	0.5106	0.3978	0.1239	0.077*	

### Atomic displacement parameters (Å^2^)

**Table:**

	*U*^11^	*U*^22^	*U*^33^	*U*^12^	*U*^13^	*U*^23^
Ag1	0.0492 (3)	0.0492 (3)	0.0444 (5)	0.02459 (16)	0.000	0.000
P1	0.0451 (8)	0.0450 (7)	0.0468 (9)	0.0208 (6)	0.0015 (6)	0.0032 (6)
P2	0.0519 (9)	0.0519 (9)	0.0387 (13)	0.0260 (4)	0.000	0.000
F1	0.124 (15)	0.082 (12)	0.163 (18)	0.076 (10)	−0.001 (18)	0.001 (14)
F2	0.16 (3)	0.12 (2)	0.17 (2)	0.038 (15)	0.014 (19)	0.048 (16)
F3	0.158 (18)	0.134 (19)	0.14 (2)	0.081 (14)	0.01 (2)	0.020 (17)
F4	0.17 (3)	0.15 (3)	0.098 (12)	0.066 (18)	0.02 (2)	−0.027 (17)
B1	0.094 (15)	0.094 (15)	0.15 (3)	0.047 (7)	0.000	0.000
B2	0.14 (2)	0.14 (2)	0.10 (4)	0.071 (12)	0.000	0.000
C1	0.048 (3)	0.045 (3)	0.054 (3)	0.024 (3)	0.005 (3)	0.006 (3)
C2	0.043 (3)	0.054 (3)	0.074 (4)	0.022 (3)	−0.003 (3)	−0.005 (3)
C3	0.053 (4)	0.060 (4)	0.087 (5)	0.019 (3)	−0.011 (3)	−0.011 (3)
C4	0.041 (3)	0.085 (5)	0.095 (5)	0.030 (4)	−0.003 (3)	0.008 (4)
C5	0.060 (4)	0.071 (4)	0.111 (6)	0.040 (4)	−0.001 (4)	−0.001 (4)
C6	0.058 (4)	0.062 (4)	0.088 (5)	0.028 (3)	−0.004 (3)	−0.004 (3)
C7	0.059 (4)	0.059 (4)	0.052 (3)	0.030 (3)	0.002 (3)	0.006 (3)
C8	0.062 (4)	0.109 (5)	0.061 (4)	0.030 (4)	0.005 (3)	0.019 (4)
C9	0.092 (6)	0.147 (8)	0.071 (5)	0.043 (5)	0.027 (5)	0.035 (5)
C10	0.105 (6)	0.138 (7)	0.054 (4)	0.065 (6)	−0.003 (5)	0.015 (5)
C11	0.087 (5)	0.096 (5)	0.061 (4)	0.054 (4)	−0.013 (4)	−0.004 (4)
C12	0.071 (4)	0.070 (4)	0.054 (4)	0.036 (3)	−0.005 (3)	−0.001 (3)
C13	0.044 (3)	0.044 (3)	0.054 (3)	0.019 (3)	0.000 (3)	0.004 (3)
C14	0.065 (4)	0.059 (4)	0.063 (4)	0.031 (3)	0.003 (3)	0.006 (3)
C15	0.084 (5)	0.052 (4)	0.092 (5)	0.040 (4)	−0.010 (4)	0.007 (4)
C16	0.074 (4)	0.058 (4)	0.100 (6)	0.037 (4)	−0.009 (4)	−0.012 (4)
C17	0.069 (4)	0.078 (5)	0.072 (4)	0.042 (4)	−0.002 (3)	−0.014 (4)
C18	0.061 (4)	0.053 (3)	0.066 (4)	0.025 (3)	0.007 (3)	0.005 (3)
C19	0.057 (3)	0.057 (3)	0.039 (3)	0.032 (3)	0.000 (3)	0.003 (3)
C20	0.079 (4)	0.074 (4)	0.061 (4)	0.051 (4)	−0.015 (3)	−0.023 (3)
C21	0.095 (5)	0.087 (5)	0.074 (5)	0.047 (4)	−0.015 (4)	−0.026 (4)
C22	0.092 (5)	0.075 (4)	0.088 (5)	0.053 (4)	0.011 (4)	−0.008 (4)
C23	0.076 (4)	0.081 (4)	0.082 (5)	0.048 (4)	0.001 (4)	0.001 (4)
C24	0.070 (4)	0.075 (4)	0.054 (4)	0.042 (3)	−0.002 (3)	−0.004 (3)

### Geometric parameters (Å, º)

**Table:**

Ag1—P2	2.633 (2)	B2—F3^viii^	1.38 (2)
Ag1—P1^i^	2.6772 (13)	B2—F3^ix^	1.38 (2)
Ag1—P1	2.6772 (13)	B2—F3^x^	1.38 (2)
Ag1—P1^ii^	2.6772 (13)	B2—F3^xi^	1.38 (2)
P1—C7	1.824 (6)	C1—C6	1.380 (7)
P1—C1	1.826 (5)	C1—C2	1.388 (7)
P1—C13	1.831 (5)	C2—C3	1.379 (7)
P2—C19^i^	1.822 (5)	C2—H2	0.9300
P2—C19^ii^	1.822 (5)	C3—C4	1.367 (8)
P2—C19	1.822 (5)	C3—H3	0.9300
F1—F2^iii^	0.97 (4)	C4—C5	1.369 (8)
F1—B1	1.37 (2)	C4—H4	0.9300
F1—F2	1.39 (5)	C5—C6	1.382 (8)
F1—F2^iv^	1.66 (2)	C5—H5	0.9300
F1—F1^iv^	1.70 (4)	C6—H6	0.9300
F1—F1^v^	1.70 (4)	C7—C8	1.386 (8)
F2—F1^vi^	0.97 (4)	C7—C12	1.398 (8)
F2—B1	1.34 (3)	C8—C9	1.409 (9)
F2—F2^iii^	1.52 (4)	C8—H8	0.9300
F2—F2^vi^	1.52 (4)	C9—C10	1.363 (10)
F2—F1^v^	1.66 (2)	C9—H9	0.9300
F3—F4^vii^	1.16 (4)	C10—C11	1.353 (9)
F3—B2	1.38 (2)	C10—H10	0.9300
F3—F4^viii^	1.42 (5)	C11—C12	1.363 (8)
F3—F3^ix^	1.59 (3)	C11—H11	0.9300
F3—F3^x^	1.59 (3)	C12—H12	0.9300
F3—F4^xi^	1.65 (3)	C13—C18	1.392 (7)
F4—F3^viii^	1.16 (4)	C13—C14	1.401 (7)
F4—F4^vii^	1.27 (4)	C14—C15	1.382 (8)
F4—F4^viii^	1.27 (4)	C14—H14	0.9300
F4—B2	1.301 (18)	C15—C16	1.376 (9)
F4—F3^vii^	1.42 (5)	C15—H15	0.9300
F4—F3^xi^	1.65 (3)	C16—C17	1.359 (8)
B1—F2^iv^	1.34 (3)	C16—H16	0.9300
B1—F2^xii^	1.34 (3)	C17—C18	1.377 (8)
B1—F2^v^	1.34 (3)	C17—H17	0.9300
B1—F2^vi^	1.34 (3)	C18—H18	0.9300
B1—F2^iii^	1.34 (3)	C19—C24	1.389 (7)
B1—F1^iv^	1.37 (2)	C19—C20	1.390 (7)
B1—F1^v^	1.37 (2)	C20—C21	1.381 (8)
B1—F1^vi^	1.37 (2)	C20—H20	0.9300
B1—F1^iii^	1.37 (2)	C21—C22	1.360 (9)
B1—F1^xii^	1.37 (2)	C21—H21	0.9300
B2—F4^viii^	1.301 (18)	C22—C23	1.374 (9)
B2—F4^vii^	1.301 (18)	C22—H22	0.9300
B2—F4^xi^	1.301 (18)	C23—C24	1.352 (8)
B2—F4^x^	1.301 (18)	C23—H23	0.9300
B2—F4^ix^	1.301 (18)	C24—H24	0.9300
B2—F3^vii^	1.38 (2)		
			
P2—Ag1—P1^i^	109.45 (3)	F1^vi^—B1—F1^xii^	76.9 (13)
P2—Ag1—P1	109.45 (3)	F1^iii^—B1—F1^xii^	76.9 (13)
P1^i^—Ag1—P1	109.49 (3)	F1—B1—F1^xii^	179.994 (18)
P2—Ag1—P1^ii^	109.45 (3)	F4^viii^—B2—F4^vii^	58.5 (15)
P1^i^—Ag1—P1^ii^	109.49 (3)	F4^viii^—B2—F4	58.5 (15)
P1—Ag1—P1^ii^	109.49 (3)	F4^vii^—B2—F4	58.5 (15)
C7—P1—C1	103.8 (2)	F4^viii^—B2—F4^xi^	121.5 (15)
C7—P1—C13	102.6 (2)	F4^vii^—B2—F4^xi^	121.5 (15)
C1—P1—C13	102.0 (2)	F4—B2—F4^xi^	180.0 (8)
C7—P1—Ag1	115.64 (18)	F4^viii^—B2—F4^x^	121.5 (15)
C1—P1—Ag1	110.91 (16)	F4^vii^—B2—F4^x^	180 (2)
C13—P1—Ag1	119.93 (17)	F4—B2—F4^x^	121.5 (15)
C19^i^—P2—C19^ii^	102.6 (2)	F4^xi^—B2—F4^x^	58.5 (15)
C19^i^—P2—C19	102.6 (2)	F4^viii^—B2—F4^ix^	180.0 (8)
C19^ii^—P2—C19	102.6 (2)	F4^vii^—B2—F4^ix^	121.5 (15)
C19^i^—P2—Ag1	115.66 (17)	F4—B2—F4^ix^	121.5 (15)
C19^ii^—P2—Ag1	115.66 (17)	F4^xi^—B2—F4^ix^	58.5 (15)
C19—P2—Ag1	115.66 (17)	F4^x^—B2—F4^ix^	58.5 (15)
F2^iii^—F1—B1	67 (3)	F4^viii^—B2—F3^vii^	51 (2)
F2^iii^—F1—F2	78 (3)	F4^vii^—B2—F3^vii^	104.3 (14)
B1—F1—F2	58.1 (14)	F4—B2—F3^vii^	64 (2)
F2^iii^—F1—F2^iv^	111 (4)	F4^xi^—B2—F3^vii^	116 (2)
B1—F1—F2^iv^	51.4 (15)	F4^x^—B2—F3^vii^	75.7 (14)
F2—F1—F2^iv^	92 (2)	F4^ix^—B2—F3^vii^	129 (2)
F2^iii^—F1—F1^iv^	71 (2)	F4^viii^—B2—F3^viii^	104.3 (14)
B1—F1—F1^iv^	51.5 (7)	F4^vii^—B2—F3^viii^	64 (2)
F2—F1—F1^iv^	109.3 (15)	F4—B2—F3^viii^	51 (2)
F2^iv^—F1—F1^iv^	49 (2)	F4^xi^—B2—F3^viii^	129 (2)
F2^iii^—F1—F1^v^	118 (3)	F4^x^—B2—F3^viii^	116 (2)
B1—F1—F1^v^	51.5 (7)	F4^ix^—B2—F3^viii^	75.7 (14)
F2—F1—F1^v^	64.0 (10)	F3^vii^—B2—F3^viii^	109.5 (10)
F2^iv^—F1—F1^v^	33.5 (15)	F4^viii^—B2—F3^ix^	75.7 (14)
F1^iv^—F1—F1^v^	78 (2)	F4^vii^—B2—F3^ix^	116 (2)
F1^vi^—F2—B1	71 (3)	F4—B2—F3^ix^	129 (2)
F1^vi^—F2—F1	130 (5)	F4^xi^—B2—F3^ix^	51 (2)
B1—F2—F1	60 (2)	F4^x^—B2—F3^ix^	64 (2)
F1^vi^—F2—F2^iii^	117 (3)	F4^ix^—B2—F3^ix^	104.3 (14)
B1—F2—F2^iii^	55.4 (9)	F3^vii^—B2—F3^ix^	70.5 (10)
F1—F2—F2^iii^	39 (2)	F3^viii^—B2—F3^ix^	180 (2)
F1^vi^—F2—F2^vi^	63 (3)	F4^viii^—B2—F3^x^	129 (2)
B1—F2—F2^vi^	55.4 (9)	F4^vii^—B2—F3^x^	75.7 (14)
F1—F2—F2^vi^	95 (2)	F4—B2—F3^x^	116 (2)
F2^iii^—F2—F2^vi^	60.000 (9)	F4^xi^—B2—F3^x^	64 (2)
F1^vi^—F2—F1^v^	76 (3)	F4^x^—B2—F3^x^	104.3 (14)
B1—F2—F1^v^	53.0 (11)	F4^ix^—B2—F3^x^	51 (2)
F1—F2—F1^v^	67 (3)	F3^vii^—B2—F3^x^	180 (2)
F2^iii^—F2—F1^v^	93.8 (19)	F3^viii^—B2—F3^x^	70.5 (10)
F2^vi^—F2—F1^v^	105.2 (11)	F3^ix^—B2—F3^x^	109.5 (10)
F4^vii^—F3—B2	61.0 (17)	F4^viii^—B2—F3	64 (2)
F4^vii^—F3—F4^viii^	58 (2)	F4^vii^—B2—F3	51 (2)
B2—F3—F4^viii^	55.5 (14)	F4—B2—F3	104.3 (14)
F4^vii^—F3—F3^ix^	110.8 (16)	F4^xi^—B2—F3	75.7 (14)
B2—F3—F3^ix^	54.7 (5)	F4^x^—B2—F3	129 (2)
F4^viii^—F3—F3^ix^	66.1 (12)	F4^ix^—B2—F3	116 (2)
F4^vii^—F3—F3^x^	71.6 (15)	F3^vii^—B2—F3	109.5 (10)
B2—F3—F3^x^	54.7 (5)	F3^viii^—B2—F3	109.5 (10)
F4^viii^—F3—F3^x^	106.9 (14)	F3^ix^—B2—F3	70.5 (10)
F3^ix^—F3—F3^x^	90 (2)	F3^x^—B2—F3	70.5 (10)
F4^vii^—F3—F4^xi^	107 (3)	F4^viii^—B2—F3^xi^	116 (2)
B2—F3—F4^xi^	50.0 (13)	F4^vii^—B2—F3^xi^	129 (2)
F4^viii^—F3—F4^xi^	95 (3)	F4—B2—F3^xi^	75.7 (14)
F3^ix^—F3—F4^xi^	41.9 (19)	F4^xi^—B2—F3^xi^	104.3 (14)
F3^x^—F3—F4^xi^	52 (2)	F4^x^—B2—F3^xi^	51 (2)
F3^viii^—F4—F4^vii^	71 (4)	F4^ix^—B2—F3^xi^	64 (2)
F3^viii^—F4—F4^viii^	121 (3)	F3^vii^—B2—F3^xi^	70.5 (10)
F4^vii^—F4—F4^viii^	60.000 (6)	F3^viii^—B2—F3^xi^	70.5 (10)
F3^viii^—F4—B2	67.9 (19)	F3^ix^—B2—F3^xi^	109.5 (10)
F4^vii^—F4—B2	60.7 (7)	F3^x^—B2—F3^xi^	109.5 (10)
F4^viii^—F4—B2	60.7 (7)	F3—B2—F3^xi^	180 (2)
F3^viii^—F4—F3^vii^	122 (3)	C6—C1—C2	117.4 (5)
F4^vii^—F4—F3^vii^	104 (3)	C6—C1—P1	118.2 (4)
F4^viii^—F4—F3^vii^	51 (3)	C2—C1—P1	124.2 (4)
B2—F4—F3^vii^	60.8 (19)	C3—C2—C1	121.2 (5)
F3^viii^—F4—F3^xi^	66.5 (14)	C3—C2—H2	119.4
F4^vii^—F4—F3^xi^	111.3 (13)	C1—C2—H2	119.4
F4^viii^—F4—F3^xi^	102 (2)	C4—C3—C2	120.3 (6)
B2—F4—F3^xi^	54.3 (9)	C4—C3—H3	119.8
F3^vii^—F4—F3^xi^	62.1 (16)	C2—C3—H3	119.8
F2^iv^—B1—F2^xii^	69.2 (18)	C3—C4—C5	119.5 (6)
F2^iv^—B1—F2^v^	69.2 (18)	C3—C4—H4	120.2
F2^xii^—B1—F2^v^	69.2 (18)	C5—C4—H4	120.2
F2^iv^—B1—F2^vi^	180.0 (15)	C4—C5—C6	120.2 (6)
F2^xii^—B1—F2^vi^	110.8 (18)	C4—C5—H5	119.9
F2^v^—B1—F2^vi^	110.8 (18)	C6—C5—H5	119.9
F2^iv^—B1—F2	110.8 (18)	C1—C6—C5	121.3 (6)
F2^xii^—B1—F2	179.995 (13)	C1—C6—H6	119.3
F2^v^—B1—F2	110.8 (18)	C5—C6—H6	119.3
F2^vi^—B1—F2	69.2 (18)	C8—C7—C12	118.9 (5)
F2^iv^—B1—F2^iii^	110.8 (18)	C8—C7—P1	122.6 (5)
F2^xii^—B1—F2^iii^	110.8 (18)	C12—C7—P1	118.3 (4)
F2^v^—B1—F2^iii^	179.994 (19)	C7—C8—C9	119.0 (6)
F2^vi^—B1—F2^iii^	69.2 (18)	C7—C8—H8	120.5
F2—B1—F2^iii^	69.2 (18)	C9—C8—H8	120.5
F2^iv^—B1—F1^iv^	62 (2)	C10—C9—C8	120.2 (7)
F2^xii^—B1—F1^iv^	42.0 (17)	C10—C9—H9	119.9
F2^v^—B1—F1^iv^	104.4 (11)	C8—C9—H9	119.9
F2^vi^—B1—F1^iv^	118 (2)	C11—C10—C9	120.3 (7)
F2—B1—F1^iv^	138.0 (17)	C11—C10—H10	119.8
F2^iii^—B1—F1^iv^	75.6 (11)	C9—C10—H10	119.8
F2^iv^—B1—F1^v^	42.0 (18)	C10—C11—C12	121.1 (7)
F2^xii^—B1—F1^v^	104.4 (11)	C10—C11—H11	119.5
F2^v^—B1—F1^v^	62 (2)	C12—C11—H11	119.5
F2^vi^—B1—F1^v^	138.0 (17)	C11—C12—C7	120.3 (6)
F2—B1—F1^v^	75.6 (11)	C11—C12—H12	119.9
F2^iii^—B1—F1^v^	118 (2)	C7—C12—H12	119.9
F1^iv^—B1—F1^v^	103.1 (13)	C18—C13—C14	118.2 (5)
F2^iv^—B1—F1^vi^	118 (2)	C18—C13—P1	118.3 (4)
F2^xii^—B1—F1^vi^	138.0 (17)	C14—C13—P1	123.4 (4)
F2^v^—B1—F1^vi^	75.6 (11)	C15—C14—C13	120.1 (6)
F2^vi^—B1—F1^vi^	62 (2)	C15—C14—H14	119.9
F2—B1—F1^vi^	42.0 (17)	C13—C14—H14	119.9
F2^iii^—B1—F1^vi^	104.4 (11)	C16—C15—C14	119.8 (6)
F1^iv^—B1—F1^vi^	179.996 (10)	C16—C15—H15	120.1
F1^v^—B1—F1^vi^	76.9 (13)	C14—C15—H15	120.1
F2^iv^—B1—F1^iii^	138.0 (17)	C17—C16—C15	120.8 (6)
F2^xii^—B1—F1^iii^	75.6 (11)	C17—C16—H16	119.6
F2^v^—B1—F1^iii^	118 (2)	C15—C16—H16	119.6
F2^vi^—B1—F1^iii^	42.0 (18)	C16—C17—C18	120.0 (6)
F2—B1—F1^iii^	104.4 (11)	C16—C17—H17	120.0
F2^iii^—B1—F1^iii^	62 (2)	C18—C17—H17	120.0
F1^iv^—B1—F1^iii^	76.9 (13)	C17—C18—C13	120.8 (6)
F1^v^—B1—F1^iii^	180.0 (11)	C17—C18—H18	119.6
F1^vi^—B1—F1^iii^	103.1 (13)	C13—C18—H18	119.6
F2^iv^—B1—F1	75.6 (11)	C24—C19—C20	118.3 (5)
F2^xii^—B1—F1	118 (2)	C24—C19—P2	118.6 (4)
F2^v^—B1—F1	138.1 (17)	C20—C19—P2	123.1 (4)
F2^vi^—B1—F1	104.4 (11)	C21—C20—C19	119.5 (6)
F2—B1—F1	62 (2)	C21—C20—H20	120.3
F2^iii^—B1—F1	42.0 (17)	C19—C20—H20	120.3
F1^iv^—B1—F1	76.9 (13)	C22—C21—C20	120.9 (6)
F1^v^—B1—F1	76.9 (13)	C22—C21—H21	119.5
F1^vi^—B1—F1	103.1 (13)	C20—C21—H21	119.5
F1^iii^—B1—F1	103.1 (13)	C21—C22—C23	119.8 (6)
F2^iv^—B1—F1^xii^	104.4 (11)	C21—C22—H22	120.1
F2^xii^—B1—F1^xii^	62 (2)	C23—C22—H22	120.1
F2^v^—B1—F1^xii^	42.0 (17)	C24—C23—C22	120.2 (6)
F2^vi^—B1—F1^xii^	75.6 (11)	C24—C23—H23	119.9
F2—B1—F1^xii^	118 (2)	C22—C23—H23	119.9
F2^iii^—B1—F1^xii^	138.0 (17)	C23—C24—C19	121.3 (6)
F1^iv^—B1—F1^xii^	103.1 (13)	C23—C24—H24	119.3
F1^v^—B1—F1^xii^	103.1 (13)	C19—C24—H24	119.3
			
P2—Ag1—P1—C7	−177.16 (19)	F3^viii^—F4—B2—F4^xi^	65 (100)
P1^i^—Ag1—P1—C7	62.9 (2)	F4^vii^—F4—B2—F4^xi^	145 (100)
P1^ii^—Ag1—P1—C7	−57.2 (2)	F4^viii^—F4—B2—F4^xi^	−145 (100)
P2—Ag1—P1—C1	65.07 (18)	F3^vii^—F4—B2—F4^xi^	−86 (100)
P1^i^—Ag1—P1—C1	−54.9 (2)	F3^xi^—F4—B2—F4^xi^	−11 (100)
P1^ii^—Ag1—P1—C1	−174.96 (18)	F3^viii^—F4—B2—F4^x^	100 (4)
P2—Ag1—P1—C13	−53.4 (2)	F4^vii^—F4—B2—F4^x^	180.000 (11)
P1^i^—Ag1—P1—C13	−173.35 (19)	F4^viii^—F4—B2—F4^x^	−110.1 (6)
P1^ii^—Ag1—P1—C13	66.6 (2)	F3^vii^—F4—B2—F4^x^	−51 (3)
P1^i^—Ag1—P2—C19^i^	57.77 (18)	F3^xi^—F4—B2—F4^x^	24 (4)
P1—Ag1—P2—C19^i^	−62.23 (18)	F3^viii^—F4—B2—F4^ix^	30 (4)
P1^ii^—Ag1—P2—C19^i^	177.77 (18)	F4^vii^—F4—B2—F4^ix^	110.1 (6)
P1^i^—Ag1—P2—C19^ii^	−62.23 (18)	F4^viii^—F4—B2—F4^ix^	180.000 (14)
P1—Ag1—P2—C19^ii^	177.77 (18)	F3^vii^—F4—B2—F4^ix^	−121 (3)
P1^ii^—Ag1—P2—C19^ii^	57.77 (18)	F3^xi^—F4—B2—F4^ix^	−46 (4)
P1^i^—Ag1—P2—C19	177.77 (18)	F3^viii^—F4—B2—F3^vii^	151.3 (19)
P1—Ag1—P2—C19	57.77 (18)	F4^vii^—F4—B2—F3^vii^	−129 (3)
P1^ii^—Ag1—P2—C19	−62.23 (18)	F4^viii^—F4—B2—F3^vii^	−59 (3)
F2^iii^—F1—F2—F1^vi^	84 (6)	F3^xi^—F4—B2—F3^vii^	75.0 (10)
B1—F1—F2—F1^vi^	13 (4)	F4^vii^—F4—B2—F3^viii^	80 (4)
F2^iv^—F1—F2—F1^vi^	−27 (4)	F4^viii^—F4—B2—F3^viii^	150 (4)
F1^iv^—F1—F2—F1^vi^	20 (6)	F3^vii^—F4—B2—F3^viii^	−151.3 (19)
F1^v^—F1—F2—F1^vi^	−46 (3)	F3^xi^—F4—B2—F3^viii^	−76.3 (9)
F2^iii^—F1—F2—B1	71 (3)	F3^viii^—F4—B2—F3^ix^	180.000 (2)
F2^iv^—F1—F2—B1	−40.6 (18)	F4^vii^—F4—B2—F3^ix^	−100 (4)
F1^iv^—F1—F2—B1	6 (2)	F4^viii^—F4—B2—F3^ix^	−30 (4)
F1^v^—F1—F2—B1	−59.2 (9)	F3^vii^—F4—B2—F3^ix^	28.7 (19)
B1—F1—F2—F2^iii^	−71 (3)	F3^xi^—F4—B2—F3^ix^	103.7 (9)
F2^iv^—F1—F2—F2^iii^	−111 (4)	F3^viii^—F4—B2—F3^x^	−28.7 (19)
F1^iv^—F1—F2—F2^iii^	−65 (2)	F4^vii^—F4—B2—F3^x^	51 (3)
F1^v^—F1—F2—F2^iii^	−130 (4)	F4^viii^—F4—B2—F3^x^	121 (3)
F2^iii^—F1—F2—F2^vi^	25 (3)	F3^vii^—F4—B2—F3^x^	180.000 (8)
B1—F1—F2—F2^vi^	−45.4 (10)	F3^xi^—F4—B2—F3^x^	−105.0 (10)
F2^iv^—F1—F2—F2^vi^	−86 (2)	F3^viii^—F4—B2—F3	−103.7 (9)
F1^iv^—F1—F2—F2^vi^	−39 (2)	F4^vii^—F4—B2—F3	−24 (4)
F1^v^—F1—F2—F2^vi^	−104.6 (15)	F4^viii^—F4—B2—F3	46 (4)
F2^iii^—F1—F2—F1^v^	130 (4)	F3^vii^—F4—B2—F3	105.0 (10)
B1—F1—F2—F1^v^	59.2 (9)	F3^xi^—F4—B2—F3	180.00 (2)
F2^iv^—F1—F2—F1^v^	18.6 (17)	F3^viii^—F4—B2—F3^xi^	76.3 (9)
F1^iv^—F1—F2—F1^v^	65 (3)	F4^vii^—F4—B2—F3^xi^	156 (4)
F1^vi^—F2—B1—F2^iv^	−110 (4)	F4^viii^—F4—B2—F3^xi^	−134 (4)
F1—F2—B1—F2^iv^	59.5 (19)	F3^vii^—F4—B2—F3^xi^	−75.0 (10)
F2^iii^—F2—B1—F2^iv^	105.2 (9)	F4^vii^—F3—B2—F4^viii^	70 (2)
F2^vi^—F2—B1—F2^iv^	180.000 (12)	F3^ix^—F3—B2—F4^viii^	−83.3 (15)
F1^v^—F2—B1—F2^iv^	−23 (3)	F3^x^—F3—B2—F4^viii^	156.6 (18)
F1^vi^—F2—B1—F2^xii^	72 (6)	F4^xi^—F3—B2—F4^viii^	−137 (3)
F1—F2—B1—F2^xii^	−119 (6)	F4^viii^—F3—B2—F4^vii^	−70 (2)
F2^iii^—F2—B1—F2^xii^	−74 (8)	F3^ix^—F3—B2—F4^vii^	−153 (2)
F2^vi^—F2—B1—F2^xii^	1 (8)	F3^x^—F3—B2—F4^vii^	87.1 (16)
F1^v^—F2—B1—F2^xii^	158 (5)	F4^xi^—F3—B2—F4^vii^	154 (3)
F1^vi^—F2—B1—F2^v^	−35 (4)	F4^vii^—F3—B2—F4	26 (3)
F1—F2—B1—F2^v^	134 (2)	F4^viii^—F3—B2—F4	−43 (3)
F2^iii^—F2—B1—F2^v^	180.005 (4)	F3^ix^—F3—B2—F4	−127 (2)
F2^vi^—F2—B1—F2^v^	−105.2 (9)	F3^x^—F3—B2—F4	113 (3)
F1^v^—F2—B1—F2^v^	52 (3)	F4^xi^—F3—B2—F4	180.00 (2)
F1^vi^—F2—B1—F2^vi^	70 (4)	F4^vii^—F3—B2—F4^xi^	−154 (3)
F1—F2—B1—F2^vi^	−120.5 (19)	F4^viii^—F3—B2—F4^xi^	137 (3)
F2^iii^—F2—B1—F2^vi^	−74.8 (9)	F3^ix^—F3—B2—F4^xi^	53 (2)
F1^v^—F2—B1—F2^vi^	157 (3)	F3^x^—F3—B2—F4^xi^	−67 (3)
F1^vi^—F2—B1—F2^iii^	145 (4)	F4^vii^—F3—B2—F4^x^	180.000 (15)
F1—F2—B1—F2^iii^	−46 (2)	F4^viii^—F3—B2—F4^x^	110 (2)
F2^vi^—F2—B1—F2^iii^	74.8 (9)	F3^ix^—F3—B2—F4^x^	27 (2)
F1^v^—F2—B1—F2^iii^	−128 (3)	F3^x^—F3—B2—F4^x^	−92.9 (16)
F1^vi^—F2—B1—F1^iv^	179.994 (11)	F4^xi^—F3—B2—F4^x^	−26 (3)
F1—F2—B1—F1^iv^	−11 (3)	F4^vii^—F3—B2—F4^ix^	−110 (2)
F2^iii^—F2—B1—F1^iv^	35 (4)	F4^viii^—F3—B2—F4^ix^	180.000 (12)
F2^vi^—F2—B1—F1^iv^	110 (4)	F3^ix^—F3—B2—F4^ix^	96.7 (15)
F1^v^—F2—B1—F1^iv^	−93.6 (19)	F3^x^—F3—B2—F4^ix^	−23.4 (18)
F1^vi^—F2—B1—F1^v^	−86.4 (19)	F4^xi^—F3—B2—F4^ix^	43 (3)
F1—F2—B1—F1^v^	82.7 (17)	F4^vii^—F3—B2—F3^vii^	92.9 (16)
F2^iii^—F2—B1—F1^v^	128 (3)	F4^viii^—F3—B2—F3^vii^	23.4 (18)
F2^vi^—F2—B1—F1^v^	−157 (3)	F3^ix^—F3—B2—F3^vii^	−60 (3)
F1—F2—B1—F1^vi^	169 (3)	F3^x^—F3—B2—F3^vii^	180.000 (2)
F2^iii^—F2—B1—F1^vi^	−145 (4)	F4^xi^—F3—B2—F3^vii^	−113 (3)
F2^vi^—F2—B1—F1^vi^	−70 (4)	F4^vii^—F3—B2—F3^viii^	−27 (2)
F1^v^—F2—B1—F1^vi^	86.4 (19)	F4^viii^—F3—B2—F3^viii^	−96.7 (15)
F1^vi^—F2—B1—F1^iii^	93.6 (19)	F3^ix^—F3—B2—F3^viii^	180.000 (9)
F1—F2—B1—F1^iii^	−97.3 (17)	F3^x^—F3—B2—F3^viii^	60 (3)
F2^iii^—F2—B1—F1^iii^	−52 (3)	F4^xi^—F3—B2—F3^viii^	127 (2)
F2^vi^—F2—B1—F1^iii^	23 (3)	F4^vii^—F3—B2—F3^ix^	153 (2)
F1^v^—F2—B1—F1^iii^	179.998 (10)	F4^viii^—F3—B2—F3^ix^	83.3 (15)
F1^vi^—F2—B1—F1	−169 (3)	F3^x^—F3—B2—F3^ix^	−120 (3)
F2^iii^—F2—B1—F1	46 (2)	F4^xi^—F3—B2—F3^ix^	−53 (2)
F2^vi^—F2—B1—F1	120.5 (19)	F4^vii^—F3—B2—F3^x^	−87.1 (16)
F1^v^—F2—B1—F1	−82.7 (17)	F4^viii^—F3—B2—F3^x^	−156.6 (18)
F1^vi^—F2—B1—F1^xii^	11 (3)	F3^ix^—F3—B2—F3^x^	120 (3)
F1—F2—B1—F1^xii^	180.00 (2)	F4^xi^—F3—B2—F3^x^	67 (3)
F2^iii^—F2—B1—F1^xii^	−134 (2)	F4^vii^—F3—B2—F3^xi^	−131 (3)
F2^vi^—F2—B1—F1^xii^	−59.5 (19)	F4^viii^—F3—B2—F3^xi^	159 (4)
F1^v^—F2—B1—F1^xii^	97.3 (17)	F3^ix^—F3—B2—F3^xi^	76 (4)
F2^iii^—F1—B1—F2^iv^	147 (4)	F3^x^—F3—B2—F3^xi^	−44 (4)
F2—F1—B1—F2^iv^	−124 (2)	F4^xi^—F3—B2—F3^xi^	22 (4)
F1^iv^—F1—B1—F2^iv^	64 (3)	C7—P1—C1—C6	−81.1 (5)
F1^v^—F1—B1—F2^iv^	−43.2 (18)	C13—P1—C1—C6	172.5 (5)
F2^iii^—F1—B1—F2^xii^	90 (4)	Ag1—P1—C1—C6	43.7 (5)
F2—F1—B1—F2^xii^	180.00 (2)	C7—P1—C1—C2	103.6 (5)
F2^iv^—F1—B1—F2^xii^	−56 (2)	C13—P1—C1—C2	−2.8 (5)
F1^iv^—F1—B1—F2^xii^	7 (2)	Ag1—P1—C1—C2	−131.6 (4)
F1^v^—F1—B1—F2^xii^	−99.5 (11)	C6—C1—C2—C3	−0.4 (8)
F2^iii^—F1—B1—F2^v^	−179.993 (15)	P1—C1—C2—C3	174.9 (5)
F2—F1—B1—F2^v^	−90 (4)	C1—C2—C3—C4	−0.2 (9)
F2^iv^—F1—B1—F2^v^	33 (4)	C2—C3—C4—C5	1.8 (10)
F1^iv^—F1—B1—F2^v^	97.2 (17)	C3—C4—C5—C6	−2.8 (10)
F1^v^—F1—B1—F2^v^	−10 (3)	C2—C1—C6—C5	−0.6 (9)
F2^iii^—F1—B1—F2^vi^	−33 (4)	P1—C1—C6—C5	−176.3 (5)
F2—F1—B1—F2^vi^	56 (2)	C4—C5—C6—C1	2.3 (10)
F2^iv^—F1—B1—F2^vi^	180.00 (5)	C1—P1—C7—C8	−22.7 (6)
F1^iv^—F1—B1—F2^vi^	−116 (3)	C13—P1—C7—C8	83.2 (6)
F1^v^—F1—B1—F2^vi^	136.8 (19)	Ag1—P1—C7—C8	−144.4 (5)
F2^iii^—F1—B1—F2	−90 (4)	C1—P1—C7—C12	161.1 (4)
F2^iv^—F1—B1—F2	124 (2)	C13—P1—C7—C12	−93.0 (5)
F1^iv^—F1—B1—F2	−173 (2)	Ag1—P1—C7—C12	39.5 (5)
F1^v^—F1—B1—F2	80.5 (11)	C12—C7—C8—C9	3.9 (10)
F2—F1—B1—F2^iii^	90 (4)	P1—C7—C8—C9	−172.2 (6)
F2^iv^—F1—B1—F2^iii^	−147 (4)	C7—C8—C9—C10	−4.2 (12)
F1^iv^—F1—B1—F2^iii^	−82.9 (17)	C8—C9—C10—C11	1.1 (13)
F1^v^—F1—B1—F2^iii^	170 (3)	C9—C10—C11—C12	2.4 (12)
F2^iii^—F1—B1—F1^iv^	82.9 (17)	C10—C11—C12—C7	−2.7 (10)
F2—F1—B1—F1^iv^	173 (2)	C8—C7—C12—C11	−0.5 (9)
F2^iv^—F1—B1—F1^iv^	−64 (3)	P1—C7—C12—C11	175.7 (5)
F1^v^—F1—B1—F1^iv^	−107 (2)	C7—P1—C13—C18	162.0 (4)
F2^iii^—F1—B1—F1^v^	−170 (3)	C1—P1—C13—C18	−90.7 (5)
F2—F1—B1—F1^v^	−80.5 (11)	Ag1—P1—C13—C18	32.2 (5)
F2^iv^—F1—B1—F1^v^	43.2 (18)	C7—P1—C13—C14	−22.0 (5)
F1^iv^—F1—B1—F1^v^	107 (2)	C1—P1—C13—C14	85.3 (5)
F2^iii^—F1—B1—F1^vi^	−97.1 (17)	Ag1—P1—C13—C14	−151.8 (4)
F2—F1—B1—F1^vi^	−7 (2)	C18—C13—C14—C15	2.6 (8)
F2^iv^—F1—B1—F1^vi^	116 (3)	P1—C13—C14—C15	−173.4 (4)
F1^iv^—F1—B1—F1^vi^	180.00 (2)	C13—C14—C15—C16	−1.7 (9)
F1^v^—F1—B1—F1^vi^	73 (2)	C14—C15—C16—C17	−0.8 (10)
F2^iii^—F1—B1—F1^iii^	10 (3)	C15—C16—C17—C18	2.3 (9)
F2—F1—B1—F1^iii^	99.5 (11)	C16—C17—C18—C13	−1.4 (9)
F2^iv^—F1—B1—F1^iii^	−136.8 (18)	C14—C13—C18—C17	−1.1 (8)
F1^iv^—F1—B1—F1^iii^	−73 (2)	P1—C13—C18—C17	175.1 (4)
F1^v^—F1—B1—F1^iii^	179.995 (15)	C19^i^—P2—C19—C24	171.7 (4)
F2^iii^—F1—B1—F1^xii^	−50 (5)	C19^ii^—P2—C19—C24	−82.0 (6)
F2—F1—B1—F1^xii^	40 (4)	Ag1—P2—C19—C24	44.9 (5)
F2^iv^—F1—B1—F1^xii^	164 (3)	C19^i^—P2—C19—C20	−8.8 (5)
F1^iv^—F1—B1—F1^xii^	−133 (4)	C19^ii^—P2—C19—C20	97.5 (4)
F1^v^—F1—B1—F1^xii^	120 (4)	Ag1—P2—C19—C20	−135.7 (4)
F3^viii^—F4—B2—F4^viii^	−150 (4)	C24—C19—C20—C21	2.8 (9)
F4^vii^—F4—B2—F4^viii^	−69.9 (6)	P2—C19—C20—C21	−176.7 (5)
F3^vii^—F4—B2—F4^viii^	59 (3)	C19—C20—C21—C22	−3.0 (10)
F3^xi^—F4—B2—F4^viii^	134 (4)	C20—C21—C22—C23	1.5 (11)
F3^viii^—F4—B2—F4^vii^	−80 (4)	C21—C22—C23—C24	0.3 (10)
F4^viii^—F4—B2—F4^vii^	69.9 (6)	C22—C23—C24—C19	−0.4 (10)
F3^vii^—F4—B2—F4^vii^	129 (3)	C20—C19—C24—C23	−1.1 (9)
F3^xi^—F4—B2—F4^vii^	−156 (4)	P2—C19—C24—C23	178.4 (5)

Symmetry codes: (i) −*x*+*y*+1, −*x*+1, *z*; (ii) −*y*+1, *x*−*y*, *z*; (iii) −*y*+1, *x*−*y*+1, *z*; (iv) *x*−*y*+2/3, *x*+1/3, −*z*+1/3; (v) *y*−1/3, −*x*+*y*+1/3, −*z*+1/3; (vi) −*x*+*y*, −*x*+1, *z*; (vii) −*y*+1, *x*−*y*+2, *z*; (viii) −*x*+*y*−1, −*x*+1, *z*; (ix) *x*−*y*+1, *x*+1, −*z*; (x) *y*−1, −*x*+*y*, −*z*; (xi) −*x*, −*y*+2, −*z*; (xii) −*x*+2/3, −*y*+4/3, −*z*+1/3.
